# Veno-venous extracorporeal CO_2_ removal for the treatment of severe respiratory acidosis

**DOI:** 10.1186/s13054-015-0769-0

**Published:** 2015-04-17

**Authors:** Matthew E Cove, William J Federspiel

**Affiliations:** Department of Respiratory and Critical Care Medicine, National University Hospital Singapore, NUHS Tower Block Level 10, Singapore, 119228 Singapore; Departments of Bioengineering and Critical Care Medicine, McGowan Institute for Regenerative Medicine, University of Pittsburgh, 3025 East Carson Street, Pittsburgh, PA 15203 USA

We read with interest the article by Karagiannidis and colleagues reporting the effects of extracorporeal CO_2_ removal (ECCO_2_R) in a pig model of severe respiratory acidosis [1]. However, their conclusion that blood flow rates between 750 and 1,000 ml/minute are necessary to correct severe acidosis using ECCO_2_R may have been biased by limitations in experimental methodology.

Firstly, the authors report CO_2_ removal rates for various blood flow rates using 19Fr and 14Fr catheters. However, the data clearly demonstrated blood recirculation using the 14Fr catheter, reducing CO_2_ removal efficiency. Although the authors mention this limitation, it is curious why the 14Fr data were presented at all, since recirculation confounds meaningful interpretation.

Secondly, a 15-minute equilibration time was used between experimental set points, but no evidence is provided that equilibrium was achieved. It is reasonable to expect equilibration within 15 minutes when the entire cardiac output participates in gas exchange, but in this study blood flow rates of only 200 to 1,000 ml/minute passed through the gas exchanger. Longer equilibration times may have resulted in continued pH correction, as demonstrated in a human ECCO_2_R pilot study using approximately 450 ml/minute blood flows [2].

Finally, this study demonstrates reductions of partial pressure of CO_2_ from 107.9 to 76.9 mmHg with blood flows of 500 ml/minute. In clinical practice this may be sufficient, a reduction from 80-85 to 60-65 mmHg in chronic obstructive pulmonary disease patients with respiratory acidosis normalized pH, allowing intubation to be avoided [2].

Although ECCO_2_R with higher blood flows clearly increases CO_2_ removal, lower flows with appropriately designed catheters may provide sufficient support for severe hypercapnic respiratory failure.

## Abbreviation

ECCO_2_R: Extracorporeal CO_2_ removal
