# Supplementary material for: Veno-venous extracorporeal CO2 removal for the treatment of severe respiratory acidosis
Source: Crit Care. 2015 Apr 17;19(1):176. doi: 10.1186/s13054-015-0769-0 (PMC4403910; doi:10.1186/s13054-015-0769-0)
Supplement: Related texts

# Reply: Author response

**Authors:** Christian Karagiannidis, Wolfram Windisch, Thomas Mueller

We are grateful for the comments to our recent manuscript [1] provided by Cove and Federspiel and we very much appreciate their effort in further elucidating the concept of ECCO_2_R. We are happy to respond to their comments as follows.

Cove and Federspiel are correct in stating that ECCO_2_R was insufficient using 14.5Fr catheters and that this was also attributable to recirculation. It was one of the intentions of our study to explicitly show this [1]. However, ECCO_2_R was insufficient in our experimental setting, too, when using 19Fr catheters at blood flow rates of 500 ml/minute while recirculation was negligible. This indicates that lower blood rates *per se* contributed to insufficient ECCO_2_R.

We agree that equilibrium for CO_2_ may not have been completed after 15 minutes, because bicarbonate is stored in slow compartments. However, in addition to the information already given in the methods section we observed that CO_2_ measures at the exhaust outlet were very stable after 15 minutes, supporting the assumption that equilibrium for CO_2_ was largely achieved.

Finally, we acknowledge the existing, albeit small scientific evidence for the clinical application of ECCO_2_R in patients. The patients in the study mentioned were far less acidotic, were mostly breathing spontaneously and were in a fairly stable condition [2]. Therefore, the results of our study setting with severely acidotic pigs are not transferrable to these conditions. To normalize severe respiratory acidosis, blood flows in the range of 750 to 1,000 ml/minute are necessary; less may be sufficient to avoid intubation in non-invasive ventilation failure under certain conditions.
